# Acute lymphoblastic leukemia cells are sensitive to disturbances in protein homeostasis induced by proteasome deubiquitinase inhibition

**DOI:** 10.18632/oncotarget.15501

**Published:** 2017-02-18

**Authors:** Magdalena Mazurkiewicz, Ellin-Kristina Hillert, Xin Wang, Paola Pellegrini, Maria Hägg Olofsson, Karthik Selvaraju, Padraig D’Arcy, Stig Linder

**Affiliations:** ^1^ Cancer Center Karolinska, Department of Oncology and Pathology, Karolinska Institute, S-171 76 Stockholm, Sweden; ^2^ Department of Medical Health Sciences (IMH), Linköping University, S-751 85 Linköping, Sweden

**Keywords:** acute lymphoblastic leukemia, proteasome, deubiquitinase, ER stress, VLX1570

## Abstract

The non-genotoxic nature of proteasome inhibition makes it an attractive therapeutic option for the treatment of pediatric malignancies. We recently described the small molecule VLX1570 as an inhibitor of proteasome deubiquitinase (DUB) activity that induces proteotoxic stress and apoptosis in cancer cells. Here we show that acute lymphoblastic leukemia (ALL) cells are highly sensitive to treatment with VLX1570, resulting in the accumulation of polyubiquitinated proteasome substrates and loss of cell viability. VLX1570 treatment increased the levels of a number of proteins, including the chaperone HSP70B’, the oxidative stress marker heme oxygenase-1 (HO-1) and the cell cycle regulator p21^Cip1^. Unexpectedly, polyubiquitin accumulation was found to be uncoupled from ER stress in ALL cells. Thus, increased phosphorylation of eIF2α occurred only at supra-pharmacological VLX1570 concentrations and did not correlate with polyubiquitin accumulation. Total cellular protein synthesis was found to decrease in the absence of eIF2α phosphorylation. Furthermore, ISRIB (Integrated Stress Response inhibitor) did not overcome the inhibition of protein synthesis. We finally show that VLX1570 can be combined with L-asparaginase for additive or synergistic antiproliferative effects on ALL cells. We conclude that ALL cells are highly sensitive to the proteasome DUB inhibitor VLX1570 suggesting a novel therapeutic option for this disease.

## INTRODUCTION

Acute lymphoblastic leukemia, ALL, constitutes ∼85 percent of all leukemias in children, with a peak incidence at 2–5 years of age [1, 2]. B-cell lineage ALL is the most common form (80–85%) with the remaining 15–20% derived from a T cell lineage [1]. As ALL progresses, leukemic blasts crowd out normal cells in the bone marrow resulting in disruptions in normal blood cell homeostasis and anemia [3]. Eventually the ALL blasts infiltrate distant organs and the disease is fatal within a few weeks if left untreated [1, 3]. Dependent on the type (i.e. B-cell or T-cell lineage), ALL cells show chromosome translocations with disruption of the genes encoding the Ig or TCR receptor and the breakpoint region [4]. A number of different fusion genes have been described, resulting in the generation of novel fusion proteins, many of which are implicated in aberrant transcriptional activation, such as the TEL/AML1 fusion prevalent in pediatric ALL cases [5]. In addition to gross genetic changes, point mutations in NOTCH, FBXW7 and JAK1 are also frequent in ALL [6, 7].

Treatment regimes generally comprise of repeated cycles of chemotherapy for a duration of ∼2 years with survival > 80% [8]. The initial step in the management of ALL is induction therapy with dexamethasone, vincristine, asparaginase with or without anthracycline [3] which is usually followed by high-dose methotrexate, cytarabine and L-asparaginase. Post-remission consolidation is most often followed by long-term maintenance with mercaptopurine and methotrexate for 2 years or longer. In particular asparaginase has been highly successful for inducing remission in acute leukemias [9]. Asparaginase exerts its antileukemic activity by converting asparagine to aspartic acid in the extracellular fluid resulting in asparagine deprivation and inhibition of cell growth. Despite the high rates of successful treatment in pediatric ALL, current ALL treatments still require improvement. Furthermore, recent publications raise concern that exposure to genotoxic ALL treatments early in life may increase the risk of secondary cancers by as much as four-fold [10].

The high level of sensitivity of myeloma cells to proteasome inhibitors has been linked to high levels of production of defective ribosomal products (DRiPs) [11, 12]. Proteasomal degradation is necessary for removal of potentially toxic misfolded proteins and also serves nutritional roles under starvation conditions through the recycling of amino acids [13, 14]. The proteasome inhibitor bortezomib has shown activity as single-agent in preclinical models of ALL [15]. Preclinical studies of ALL cells demonstrated that bortezomib is synergistic with dexamethasone and additive when combined with L-asparaginase, vincristine, doxorubicin, and cytarabine [16]. Furthermore, the combination of bortezomib and chemotherapy was reported to show promising results in B-precursor ALL [15, 17].

Proteasomal deubiquitinases (DUBs) are required for proper proteasome degradation of polyubiquitinated substrates by removing bulky ubiquitin chains. The small molecule b-AP15 and its optimized lead VLX1570 interfere with the proteasomal degradation by inhibiting the activities of the proteasomal DUBs USP14 and UCHL5 [18–20]. These drugs induce gene expression profiles characteristic of proteasome inhibitors [18, 21] and the primary mechanism of cell death induction is tightly linked to proteasome inhibition [22]. VLX1570 and low-dose dexamethasone is currently in Phase I for relapsed or relapsed and refractory multiple myeloma (NCT02372240). In this study we investigate that effect of proteasome DUB inhibition on the growth and survival of ALL cells.

## RESULTS

### ALL cells are sensitive to the proteasome deubiquitinase inhibitor VLX1570

**Figure 1:**
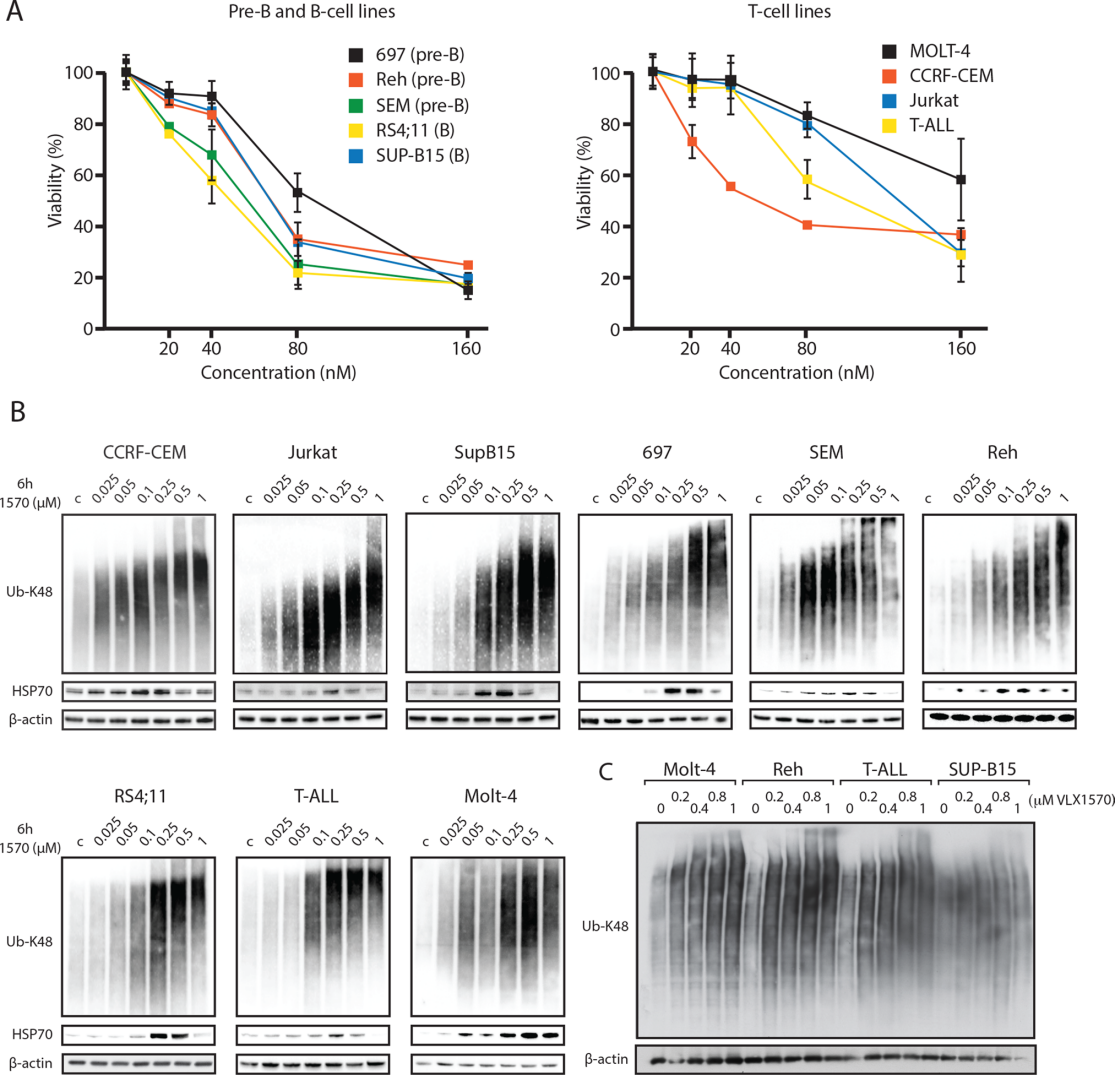
(**A**) ALL cells are sensitive to VLX1570. Pre-B, B-cell and T-cell ALL lines were incubated in the indicated concentrations of VLX1570 for 72 hours and viability was determined by the MTT assay. (**B**) ALL cell lines were exposed to the indicated concentrations of VLX1570 for 6 hours. Cell extracts were prepared and subjected to immunoblotting using antibodies to K48-linked ubiquitin, HSP70B’ and β-actin as indicated. HSP70B’ is the inducible form of Hsp70 (HSPA6). (**C**) Four ALL cell lines with different sensitivities to VLX1570 were treated for 6 h with the indicated drug concentrations, extracts prepared and transferred to the same filter. Note that although the accumulation of polyubiquitinated proteins was dose-dependent, there was no correlation between the extent of accumulation and VLX1570 sensitivity (MOLT-4 IC_50_ 180 nM; Reh IC_50_ 68 nM; T-ALL IC_50_ 100 nM; SUP-B15 IC_50_ 68 nM).

We investigated the effect of the proteasome deubiquitinase (DUB) inhibitor VLX1570 on the viability of 9 different ALL cell lines over 72 hours using the MTT viability assay. Seven of the cell lines showed IC_50_ values in the range 50–100 nM, whereas two cell lines (Jurkat and MOLT-4, both T-cell ALL lines) were slightly less sensitive (120 and 180 nM respectively) (Figure 1A). The level of sensitivity of ALL B-cell lines to VLX1570 is similar to that previously found in multiple myeloma cells [19], which are generally classified as sensitive to inhibitors of the ubiquitin-proteasome system (UPS), and higher than that of melanoma and colon cancer cells [21]. Inhibition of proteasome ubiquitin processing following VLX1570 treatment results in the accumulation of high molecular weight polyubiquitinated substrates in cells [19, 21]. We indeed found a dose-dependent increase in high-molecular polyubiquitinated proteins in ALL cells following exposure to VLX1570 (Figure 1B). The increases in polyubiquitinated proteins occurred at drug concentrations that reduced the number of viable cells (50 – 100 nM), consistent with the notion that the growth inhibitory effect of the drug is due to UPS inhibition. The extent of accumulation of misfolded protein substrates in VLX1570-exposed cells was previously found to be associated with cytotoxicity [23]. We therefore examined whether accumulation of polyubiquitinated proteins is correlated to the drug sensitivity of ALL cell lines but did not observe any such relationship (Figure 1C).

In general cells with high protein turnover rates respond to decreased UPS flux via the induction of chaperones to counteract the accumulation of misfolded proteins. We indeed found that VLX1570 increased the expression of the inducible form of Hsp70 (HSP70B´) in all ALL cell lines tested (Figure 1B). Induction of HSP70B´ was generally observed at drug concentrations that induced the accumulation of polyubiquitin. At higher concentrations of VLX1570 (0.5 to 1 μM), the induction of HSP70B was less prominent. This phenomenon occurred over the first 6 hours of treatment at a point where no cytotoxicity was observed.

### Characterization of alterations in protein expression in ALL cells exposed to VLX1570

**Figure 2:**
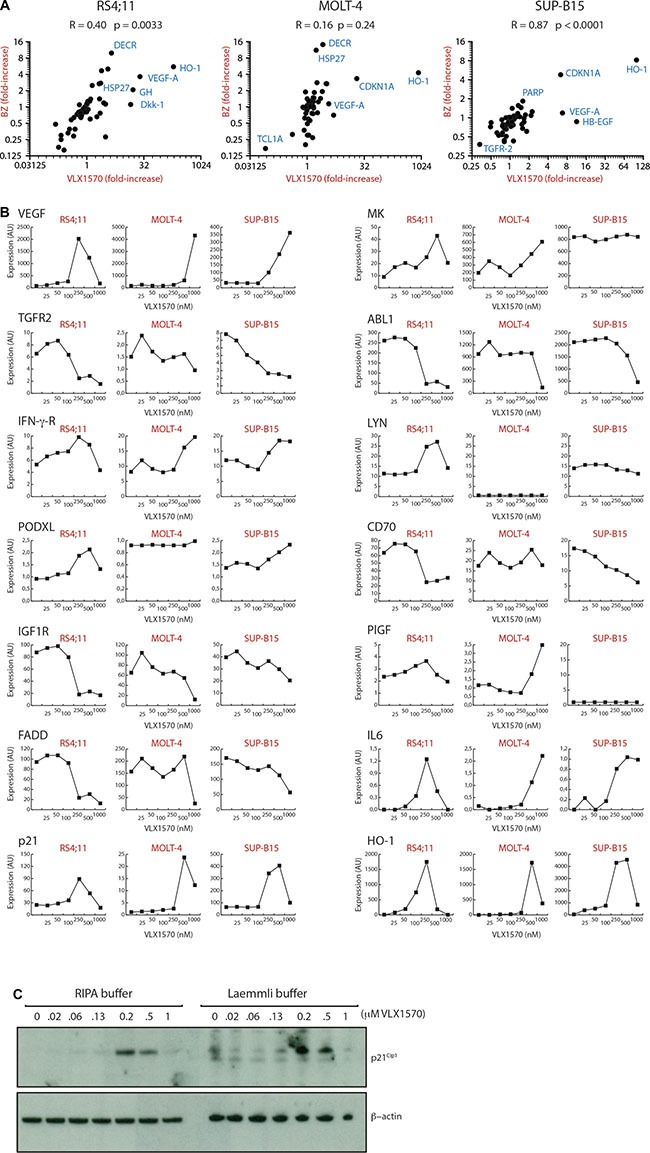
(**A**) Alterations in protein expression by VLX1570 and bortezomib. Cells were exposed to 250 nM (RS4;11) or 500 nM VLX1570 (MOLT-4, SUP-B15) or 100 nM bortezomib and the expression of 184 proteins was examined by a multiplex immunoassay (ProSeek^™^). Seventy of these were detected in all cell lines; Pearson correlation coefficients are shown as well as *p*-values calculated in Graph Prism. Markers: HO-1: heme oxygenase; Dkk-1, dickkopf-related protein; HSP27, heat shock protein 27; GH, growth hormone; VEGF-A, vascular endothelial growth factor A; DECR, 2,4-dienoyl-CoA-reductase 1 (mitochodrial); CDKN1A, p21^Cip1^, cyclin dependent kinase inhibitor 1A; HB-EGF, Heparin-binding EGF-like growth factor; TGFR-2 transforming growth factor receptor 2, PARP, Poly(ADP-ribose) polymerase. (**B**) Expression of 14 proteins in ALL cells exposed to increasing concentrations of VLX1570. The analysis was explorative to search for markers and single samples were used at each concentration. Expression is in arbitrary units according to ProSeek manual. Markers: VEGF-A, vascular endothelial growth factor A; TGFR1, transforming growth factor receptor 1; IFN-γ-R, interferon gamma receptor; PODXL, Podocalyxin-like protein 1; IGF1R, insulin growth factor 1 receptor; FADD, Fas-Associated protein with Death Domain; p21, p21Cip1; MK, midkine; ABL1, Abelson kinase 1; LYN, Lck/Yes-related novel protein tyrosine kinase; CD70; PlGF, placental growth factor; IL6, interleukin 6; HO-1: heme oxygenase. (**C**) Analysis of p21^Cip1^ expression using soluble (RIPA buffer) and total protein extracts (Laemmli buffer). MOLT-4 cells were exposed to increasing concentrations of VLX1570 and processed for western blotting using antibodies to p21^Cip1^ and β-actin.

Previous proteomics studies have shown only minor changes in protein expression upon proteasome inhibition, generally up-regulations in the order < 4-fold [24, 25]. To build upon this we used a proximity antibody-based extension multiplex assay (ProSeek^™^, [26]) to examine the expression of 184 markers in 3 different ALL cell lines (RS4;11, MOLT-4 and SUP-B15) following exposure to VLX1570. A common set of 70 proteins was detected in all three cell lines with variable levels of expression of the remaining markers. VLX1570 and the 20S proteasome inhibitor bortezomib (BZ) induce similar changes in the expression of these proteins in RS4;11 and SUP-B15 cells (*p* = 0.0033 and *p* < 0.0001, respectively), whereas no significant correlation was observed in MOLT-4 cells (Figure 2A). The most strongly induced protein was HO-1 (heme oxygenase), an NRF2-induced protein and a marker of oxidative stress [27]. The increased expression of HO-1 is consistent with earlier findings of induction of oxidative stress by b-AP15 and VLX1570 [22]. VEGF-A and CDKN1A (p21^Cip1^) were also induced in all 3 cell lines. The induction of HO-1 and p21^Cip1^ was validated in independent experiments and by Western blotting (see below). Unexpectedly, the expression of a number of proteins decreased following drug exposure. In RS4;11 cells the expression of 11 proteins (ABL1, CD70, FADD, hK8, IGF1R, IL-1ra, IL-14, IL-16, NEMO, PAR-1, TGFR-2) decreased by > 2-fold following treatment with 320 nM VLX1570.

Decreases in the levels of protein markers following exposure to VLX1570 is not consistent with previous observations of the effects of proteasome inhibitors [24]. Dose-response experiments showed that decreases in expression either occurred at moderate drug concentrations (0.2 μM in RS4;11 cells, TGFR1/2, FADD and ABL1) or were observed at concentrations of 0.5 to 1 μM and often preceded by increased expression (HO-1, p21^Cip1^) (Figure 2B). Decreased expression at high VLX1570 concentrations was primarily observed in sensitive RS4;11 cells whereas this effect was less pronounced in the less sensitive MOLT-4 cell line. We wondered whether the decreases in protein expression observed at high VXL1570 concentrations could be explained by inclusion of proteins in insoluble complexes not detectable using non-denaturing conditions for lysate preparation (used for the ProSeek assay and preparation of lysates for western blotting). To address this question, we exposed MOLT-4 ALL cells to increasing concentrations of VLX1570 and examined p21^Cip1^ in both soluble fractions and total cell lysates. We did not, however, observe any difference between the different lysate preparation methods suggesting that the discrepancies in protein levels are not due to sequesterization (Figure 2C).

### Discordant pattern of accumulation of polyubiquitinated proteins and induction of eIF2α phosphorylation in ALL cells exposed to VLX1570

**Figure 3:**
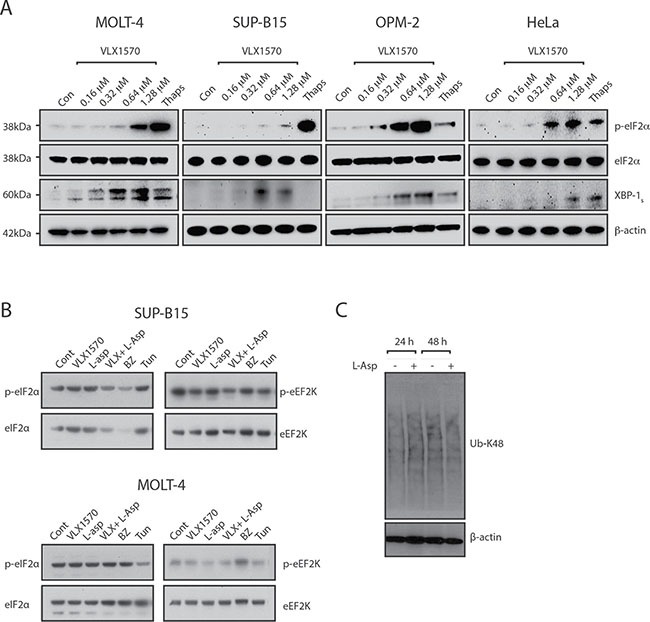
Induction of ER stress by VLX1570 (**A**) ALL (MOLT-4 and SUP-B15) and non-ALL (OPM-2 and HeLa) cells were treated with DMSO or different concentrations of VLX1570 for 6 hours. Thapsigargin was used at 10 μM for 6 hrs. Protein lysates were subjected to immunoblotting with antibodies to p-eIF2α, eIF2α, XBP-1_s_ or β-actin. (**B**) ALL (MOLT-4 and SUP-B15) cells were treated with IC_50_ concentrations of VLX1570 and/or L-Asp or bortezomib (BZ) for 6 hours. Protein lysates were subjected to immunoblotting with antibodies to p-eIF2α, eIF2α, p-eEF2K or eEF2K. Tunicamycin is an inhibitor of protein glycosylation which in distinction of thapsigargin did not induce p-eIF2α in most of the ALL cell lines (with the exception of Reh, See Supplementary Figure 1). (**C**) Analysis of polyubiquitin accumulation in RS4;11 cells following exposure to L-Asparaginase.

b-AP15 and VLX1570 induce ER stress in various cancer cell lines [22, 28–30]. ER stress is considered as an important response to proteasome inhibition, resulting in translational attenuation to prevent accumulation of misfolded proteins [31, 32]. In order to assess ER stress we examined phosphorylation of eIF2α and induction of the spliced form of X-box binding protein 1 (XBP1_S_) in SUP-B15 and MOLT-4 cells following exposure to VLX1570. Induction of both markers was observed, with the appearance of XBP1_S_ at lower drug concentrations than those that induced eIF2α phosphorylation (Figure 3A). Induction of eIF2α phosphorylation was weaker compared to that observed with thapsigargin, a well characterized SERCA pump inhibitor. Importantly, no induction of eIF2α phosphorylation was observed at drug concentrations sufficient to induce polyubiquitin accumulation and reduce cell viability. We extended the analysis to the remaining cell lines in the panel but did not observe any changes in phosphorylation of eIF2α at the pharmacologically relevant concentration of 0.16 μM VLX1570 (Figure 3B; Supplementary Figure 1). These findings demonstrate a discordance between the induction of polyubiquitin accumulation and eIF2α phosphorylation in VLX1570-exposed ALL cell lines.

The limited induction of eIF2α phosphorylation by VLX1570 in ALL cells prompted us to extend our studies to cells of non-ALL origin (OPM-2 myeloma and HeLa cells). eIF2α phosphorylation was observed at VLX1570 concentrations close to IC_50_ in both cell lines (OPM-2: 0.13 μM; HeLa: 0.68 μM) and was in fact stronger compared to thapsigargin (Figure 3A). We conclude, therefore, that VLX1570 is able to induce eIF2α phosphorylation in non-ALL cells.

ALL cells are known to be particularly sensitive to asparagine starvation. Treatment with L-asparaginase (L-asp) is known to induce eIF2α phosphorylation via the kinase GCN2 [33, 34]. We wondered whether the combination of VLX1570 and L-Asp would lead to induction of eIF2α phosphorylation. We did not, however, observe any effect on phosphorylation of eIF2α by the combination between VLX1570 and L-Asp in any of the cell lines in the panel (Figure 3B, Supplementary Figure 1). We finally found that L-asp treatment does not lead to increased accumulation of misfolded proteins in exposed ALL cells (Figure 3C).

eEF2K phosphorylates and inhibits eukaryotic elongation factor 2, to slow down the elongation stage of protein synthesis providing protection against nutrient starvation and proteotoxicity [35, 36]. We hypothesized that eEF2K phosphorylation may increase in cells exposed to VLX1570 and/or L-Asp but, in fact, observed a decreased eEF2K phosphorylation following treatment with VLX1570 in SUP-B15 cells (Figure 3B, Supplementary Figure 1). Decreased eEF2K phosphorylation was also observed by the combination of VLX1570 and L-Asp in Reh and SEM cells (Supplementary Figure 1).

We conclude that our experiments did not suggest important roles for eIF2α or eEF2K in the response to VLX1570 at concentrations that induce polyubiquitin accumulation and reduce ALL cells viability.

### Reduction of protein synthesis does not correlate to eIF2α phosphorylation or VLX1570 sensitivity

**Figure 4:**
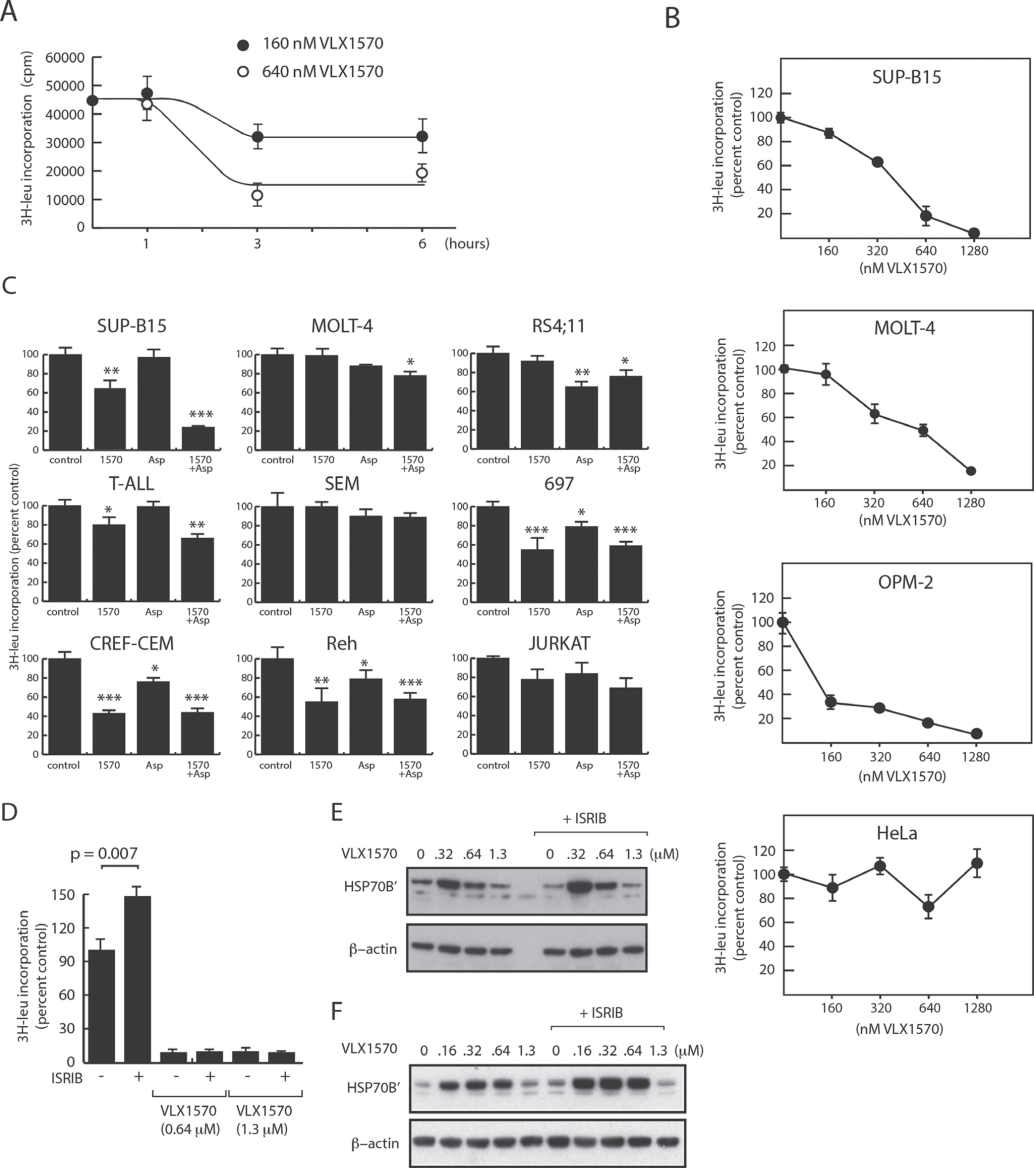
Effects of VLX1570 and L-asparaginase on protein synthesis [^3^H]-leucine incorporation into acid precipitable material was determined in cells exposed to drugs as indicated. (**A**) Time-dependent decrease in [^3^H]-leucine incorporation following exposure of SUP-B15 cells to VLX1570. (**B**) Dose-response of [^3^H]-leucine incorporation in ALL and non-ALL cell lines (6 hours drug exposure, [^3^H]-leucine added during the last hour). (**C**) [^3^H]-leucine incorporation in different ALL cell lines exposed to 0.16 μM VLX1570 and/or L-Asp for 6 hours ([^3^H]-leucine added after 5 h). Shown are average incorporation +/− S.E.M. from 5 samples. (**D**) Effect of Integrated Stress Response inhibitor (ISRIB; 0.2 μM) on [^3^H]-leucine incorporation in OPM-2 cells. (**E**, **F**) Effect of ISRIB on HSP70B’ expression in OPM-2 cells (E) and MOLT-4 cells (F). Cells were exposed to the indicated concentrations of VLX1570 in the presence or absence of 0.2 μM ISRIB. Proteins were extracted using RIPA buffer and processed for western blotting.

We hypothesized that the weak induction of eIF2α phosphorylation by VLX1570 will lead to continued protein synthesis during conditions of proteasome inhibition and to disruptions in protein homeostasis. In order to examine protein synthesis we measured total [^3^H]-leucine incorporation into the acid precipitable fraction of treated cells. Whereas no effect was observed in SUP-B15 cells at 1 h of drug incubation (using 0.16 μM and 0.64 μM VLX1570), [^3^H]-leucine incorporation decreased following 3 and 6 hours incubation (Figure 4A). These decreases were not due to decreased cell viability. Analysis of dose-response showed a gradual decrease in [^3^H]-leucine incorporation in SUP-B15 and MOLT-4 cells with increasing VLX1570 concentrations (Figure 4B). These decreases in [^3^H]-leucine incorporation did not correlate to eIF2α phosphorylation (Figure 3A). The rate of protein synthesis was more severely affected in VLX1570-sensitive SUP-B15 cells compared to less sensitive MOLT-4 cells (Figure 4B). At VLX1570 concentrations of 1.3 μM, [^3^H]-leucine incorporation was < 5% of control in SUP-B15 cells and ∼20% in MOLT-4 cells, possibly explaining the low accumulation of proteins such as HO-1 and p21^Cip1^ at this concentration. We measured the effect of VLX1570 at a concentration (0.16 μM) that reduces the number of viable cells and induces the accumulation of polyubiquitinated proteins in all ALL cell lines in the panel. Overall [^3^H]-leucine incorporation was 71 + 24 % (mean + S.D.) in drug-exposed cells compared to control (Figure 4C). The sensitivity of ALL cells to VXL1570 in viability assays did not correlate to the pattern of decreased protein synthesis. Thus, protein synthesis was weakly or not affected by VLX1570 in both resistant (MOLT-4, Jurkat) and sensitive (SEM and RS4;11) cell lines.

We extended our analysis of the effect of VLX1570 on protein synthesis to the two non-ALL cell lines studied above (OPM-2 and HeLa cells). [^3^H]-leucine incorporation in HeLa cells was not affected by VLX1570 at any concentrations tested even though phosphorylation of eIF2α was observed at 0.64 and 1.3 μM. This defective protection response was not associated with high drug sensitivity (IC_50_:0.68 μM). In contrast, [^3^H]-leucine incorporation in OPM-2 cells (IC_50_:0.13 μM) was reduced by > 50% at 0.16 μM VLX1570 and further reduced at higher drug concentrations. This pattern was not consistent with the dose-response to eIF2α phosphorylation and with the initial hypothesis that decreased protein synthesis would confer protection to VLX1570 cytotoxicity.

Our observations raised questions with regard to the role of eIF2α phosphorylation in the repression of protein synthesis in VLX1570-exposed cells. We examined the effect of ISRIB (Integrated Stress Response inhibitor), a small molecule that overcomes the inhibitory effect of eIF2α phosphorylation on translation [37, 38]. OPM-2 cells were used in this experiment due to strong eIF2α phosphorylation in this cell line. We found that ISRIB increased the basal level of protein synthesis in OPM-2 cells, but failed to alleviate the reduction in protein synthesis induced by VLX1570 (Figure 4D). Furthermore, the weaker induction of HSP70B’ observed at VLX1570 concentrations > 0.6 μM was not alleviated by ISRIB (Figure 4E). A similar result was obtained using MOLT-4 ALL cells (Figure 4F). These findings suggest that the repression of protein synthesis by VLX1570 occurs via an eIF2α-independent mechanism.

### Therapeutic effects by the combination of proteasome DUB inhibition and amino acid depletion

Our data show that protein synthesis in ALL cells is only weakly, or in some instances not at all, affected by proteasome inhibition. Continued protein synthesis during conditions of proteasome inhibition would be detrimental to cellular protein homeostasis due to the continuous synthesis of misfolded proteins and a reduction in amino acid recycling [13, 39]. ALL cells are particularly sensitive to disturbances in amino acid homeostasis and L-asparaginase (L-asp) therapy; an approved treatment option for ALL [40]. Treatment with L-asp (IC_50_ concentration) resulted in a weak but significant reduction in total protein synthesis (82 + 10% to control in the cell line panel; *p* = 0.0007, paired *t*-test). The effect of the combination with VLX1570 was generally modest (VLX1570: 71%; VLX1570 + L-asp: 62% of untreated control) with the exception of SUP-B15 cells where protein synthesis was decreased to ∼20% of the control. There was no apparent correlation between the effects of L-asp and VLX1570 on protein synthesis in the different cell lines tested (Pearson correlation coefficient = - 0.1; *p* = 0.82), suggesting that the mechanisms of protein synthesis reduction were distinct between the two types of treatments.

**Figure 5:**
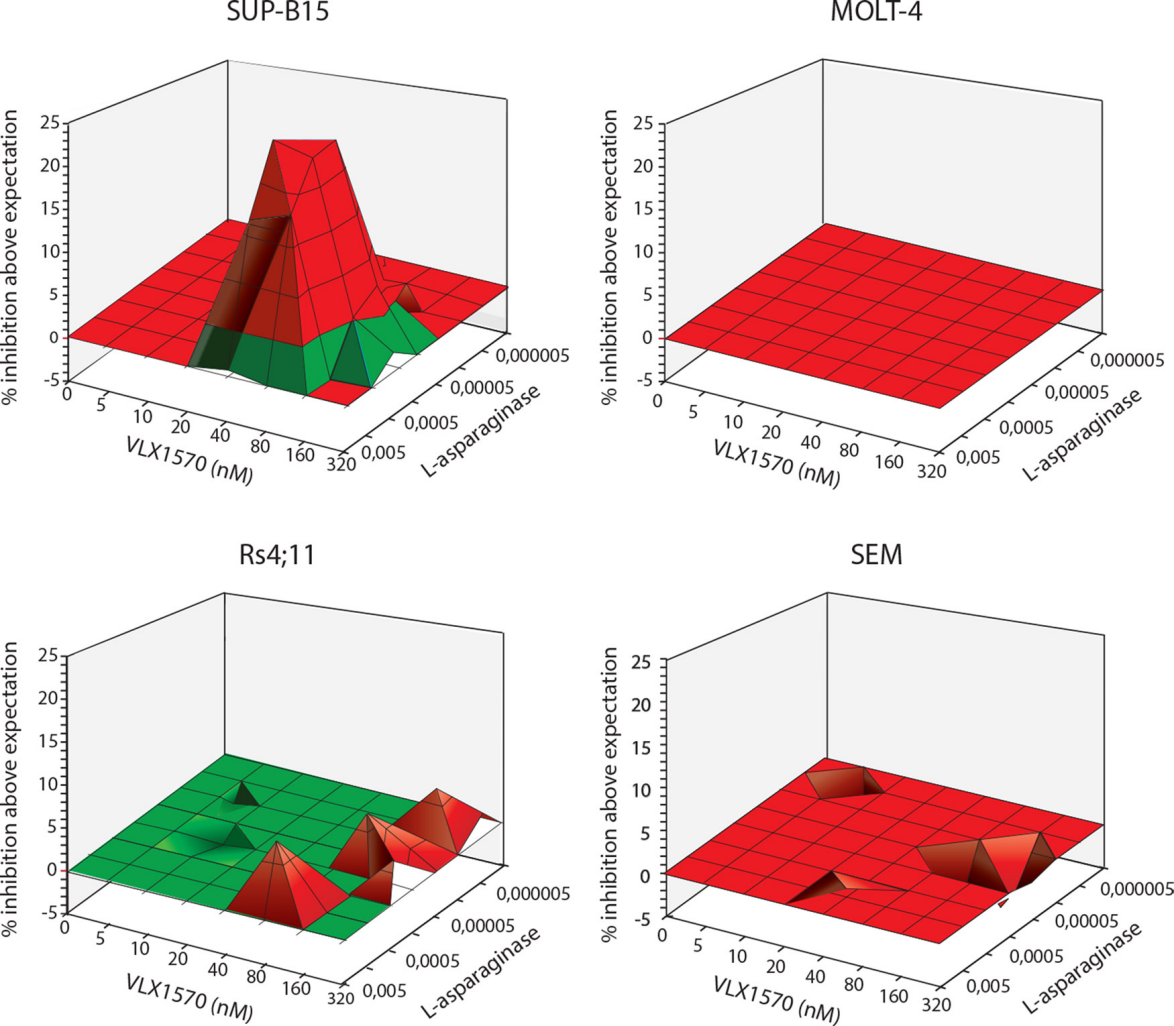
Analysis of combinatory effects of VLX1570 and L-Asp on ALL cell viability Cells were exposed to the indicated drug concentrations and viability was determined by MTT assay after 72 hours. MacSynergy software ([41]https://www.uab.edu/medicine/peds/macsynergy) was used to calculate the efficiency of drug combinations to reduce cell viability. Synergy plots generated by the MacSynergy^™^ II software reflect the difference between experimentally determined results and the theoretical drug interactions, calculated from the dose response curves for each drug individually. The resulting plot appears as a flat surface for an additive effect, peaks indicate synergy and depressions indicate antagonism. We observe additive effect in 3 cell lines (MOLT4, RS4;11 and SEM) and synergy was observed in SUP-B15 cell line. The log volume of the synergy plot of SUP-B15 cells was 23.2, a value described as strong synergy [41]. No antagonistic effect was observed in the tested cell lines.

We examined whether combining VLX1570 and L-asp would result in synergistic effects on ALL cell viability. These experiments were evaluated by a 3D surface approach where the levels of synergy between two drugs are indicated by peaks [41]. The results showed additive effects between VLX1570 and L-asp in 3 of the ALL cells tested (Figure 5). Interestingly, VLX1570 and L-asp showed strong significant synergistic effects in SUP-B15 cells (Figure 5). We considered the possibility that pre-treatment with L-asp would sensitize cells to subsequent exposure to VLX1570. This was found not to be the case.

## DISCUSSION

Bortezomib is a clinically approved inhibitor of the enzymatic activities of the 20S proteasome primarily used for treatment and management of multiple myeloma. Previous studies have shown that bortezomib displays *in vitro* activity in ALL and *in vivo* ALL xenograft models [42]. Phase II clinical trials in ALL patients have shown encouraging results suggesting that the UPS is indeed a viable target in this disease [15, 17]. An alternative approach to blocking proteasome processing is to block upstream 19S proteasome deubiquitinase (DUB) activity [43]. In this investigation we report that a panel of ALL cell lines are sensitive to the proteasome DUB inhibitor VLX1570 currently in clinical trials for multiple myeloma [NCT 02372240] and show a similar degree of sensitivity as myeloma cells (median IC_50_ 83 nM for ALL cells, 74 nM for myeloma cells [19, 21]). This level of sensitivity is much higher when compared to solid tumor cells such as colon carcinoma and melanoma cells (∼500 nM) [21]. Myeloma cells in particular are believed to be sensitive to proteasome inhibition due to high rates of immunoglobulin chain synthesis [11], resulting in the rapid accumulation of misfolded proteins during conditions of proteasome stress.

In ALL cells the sensitivity to VLX1570 was associated with the accumulation of polyubiquitinated proteins at drug concentrations (50–100 nM) that reduced the number of viable cells. We previously reported that b-AP15 and VLX1570 induce ER stress in multiple myeloma and cancer cells derived from solid tumors [22, 29]. Interestingly, in ALL cells we did not detect phosphorylation of eIF2α at drug concentrations sufficient to induce polyubiquitin accumulation and reduce cell viabilty. In MOLT-4 cells, eIF2α phosphorylation was observed at ∼1 μM (a VLX1570 concentration almost > 5-fold IC_50_) but was barely detectable in SUP-B15 cells at this concentration. The SERCA inhibitor thapsigargin elicited eIF2α phosphorylation in both cell lines showing that the pathway is not defective. It is well established that translation is generally controlled at the level of initiation [44]. The pattern of protein synthesis repression by VLX1570 did not, however, correlate with the pattern of eIF2α phosphorylation, neither in the ALL cell lines nor the myeloma cell line OPM-2. Furthermore, protein synthesis was unaffected by VLX1570 in HeLa cells despite induction of eIF2α phosphorylation. This latter observation is consistent with a previous report showing insensitivity of HeLa cell protein synthesis to induction of eIF2α phosphorylation [45]. To further address this question we utilized ISRIB, a small molecule shown to revoke the inhibitory effect of eIF2α phosphorylation on translation [37, 38]. Although ISRIB increased the basal level of protein synthesis, it did not alleviate the repression of protein synthesis by VLX1570, consistent with our other observations. Since not only initiation is important for the control of mRNA translation we examined the possibility that eEF2K may be involved in regulating protein synthesis in VLX1570-exposed cells. eEF2K is regulated by calcium/calmodulin- and mTOR- signaling and protects cells against nutrient starvation [35]. We did not, however, observe any increase in the phosphorylation of eEF2K in VLX1570-exposed cells. Although we can not at this point explain the effects of VLX1570 on protein synthesis we note that a number of chemotherapeutic drugs have been reported to affect ribosome biogenesis by mechanisms that are currently unclear [46]. Further studies will be required to elucidate the exact mechanism of action of VLX1570 on protein synthesis.

When VLX1570 was combined with L-asparaginase, protein synthesis was further suppressed in SUP-B15 cells. This effect could not be explained by eIF2α or eEF2K phosphorylation. Interestingly, however, VLX1570 and L-asparaginase induced a strong synergistic effect on the viability of SUP-B15 cells. Although the mechanism underlying this synergistic effect is unclear, the possibility to combine proteasome inhibition with L-asparaginase treatment deserves further studies.

The expression level of a number of proteins was altered by VLX1570. Increases in p21^Cip1^ and heme oxygenase (HO-1) have previously been documented [18, 19, 22] and presumably arise due to reduced UPS activity and increased levels of oxidative stress, respectively [27, 47]. HO-1 converts heme to form biliverdin and is most strongly expressed in the spleen under physiological conditions. The increase in HO-1 was quite strong (2 - 3 orders of magnitude) and this protein is a candidate pharmacodynamic marker for clinical studies involving VLX1570. At VLX1570 concentrations of ∼1 μM, p21^Cip1^ and HO-1 levels increases were less pronounced. This phenomenon was unlikely due to protein aggregation but rather due to a block in protein synthesis. A similar phenomenon of weak inductions at high VLX1570 exposures was found for HSP70B’ (Figure 1B). We conclude that decreased protein synthesis during conditions of proteasome blocking may not necessarily be viewed exclusively as a protective response by reducing the load of misfolded proteins, but may also abrogate the synthesis of chaperones, cell cycle regulators and proteins protecting against oxidative stress.

We conclude that ALL cells display *in vitro* sensitivity to VLX1570 in the same range as multiple myeloma cells. VLX1570 induced ER stress in ALL cells, but this response occurred at higher drug concentrations and did not correlate with reduction in cell viability. ALL cells are asparagine auxotrophs and we found that VLX1570 could be combined with L- asparaginase to generate additive or even synergistic effects on cell viability. We did not expect such effects at the initiation of this study–asparagine starvation may rather be cause antagonistic effect based on induction of eIF2α phosphorylation, reduction of protein synthesis and lower levels of proteotoxicity. Our results emphasize the importance of examination the effect of therapeutics at relevant drug concentrations.

## MATERIALS AND METHODS

### Materials

b-AP15 and VLX1570 were synthesized by OnTarget Chemistry AB (Uppsala, Sweden). ISRIB was obtained from Sigma Aldrich. Anti-β actin (AC-15), anti-HSPA6 (HPA028549) (Sigma Aldrich, St Louis, MO); anti-Ubiquitin K48 (Apu2) (Millipore); anti-Heme Oxygenase 1 (610712), anti PARP (556362), anti-phospho eIF2α (9721), anti-eIF2α (2103), anti-phospho EF2K (Ser 366) (3191), anti-EF2K (4153) all from Cell Signaling Technologies.

### Cell culture

ALL cells were maintained in RPMI-1640 medium supplemented with 10% fetal bovine serum and 1% penicillin/streptavidin. ALL cells were obtained from the group of Dan Grandér at the Karolinska Institute. All cells were maintained at 37°C in 5% CO_2_.

### Western blot analysis

For ubiquitin analysis, cell extract proteins were resolved by Tris-Acetate PAGE gels (Invitrogen, Carlsbad, CA) and transferred onto a polyvinylidene difluoride (PVDF) membrane for western blotting.

### Cell viability assay

Cell viability was monitored either by the MTT (3-(4,5-dimethylthiazol-2-yl)-2,5-diphenyltetrazolium bromide) assay or by phase contract microscopy. For the MTT assay, cells were suspended at 5 × 10^5^ cells/ml, and 100 μl aliquots were dispended into 96-well microtiter plates and exposed to drugs as described using DMSO control. At the end of incubations, 10 μl of a stock solution of 5 mg/ml MTT (3-(4,5-dimethylthiazol-2-yl)-2,5-diphenyltetrazolium bromide), was added into each well, and the plates were incubated 4 hours at 37°C. Formazan crystals were dissolved with 100 μl of 10% SDS/10 mM HCl solution overnight at 37°C. Absorbance was measured using an ELISA plate reader (Labsystems Multiscan RC) at 590 nm. Short term viability was assessed by phase contrast microscopy.

### Protein synthesis

Protein synthesis was measured by incorporation of Leucine L- (4,5-^3^H ) (NET1166005MC ) obtained from Perkin Elmer (USA, Waltham, MA). Prior to the treatment ALL cells were cultured in RPMI-1640 growth media with 10% FBS and 1% penicillin/streptavidin. Cells were treated for 5h with DMSO, 160 nM, 320 nM, 640 nM, 1280 nM of VLX1570, or for 3h with 160 nM VLX1570, 0,001 U L-asparaginase and combination of the two drugs. Subsequently cells were washed with PBS and RPMI-1640 media without L-leucine and 1μCi/mL Leucine L-(4,5-^3^H) was added for 1h. Cell suspensions were placed on Whatman GF/C microfiber filters and proteins were precipitated with ice-cold 5% trichloroacetic acid (TCA) (Sigma Aldrich). Filters were washed twice with 5% TCA, once with 99,5 % ethanol and left to dry. Radioactivity was measured with liquid scintillation counter. Incorporation increased linearly over time (15 – 60 minutes).

### ProSeek

ALL cells/ Sample preparation: The cells were washed once with cold PBS. 40 ul of RIPA lysis buffer was added to each sample and placed on ice for 30 minutes. All samples were centrifuged at 12000 g for 15 minutes and the protein concentrations were measured in the supernatant. 20 μl of each sample with a concentration of 1 μg/ μl was added to a 96-well PCR plate (Sarstedt #72.1979.202) and sealed with adhesive film and sent to OLINK Proteomics (Uppsala, Sweden) for analysis using ProSeek^®^ multiplex-proximity ligation assays (using CVDII and ONCII plates).

## SUPPLEMENTARY MATERIALS FIGURE


