# Supplementary material for: Acute lymphoblastic leukemia cells are sensitive to disturbances in protein homeostasis induced by proteasome deubiquitinase inhibition
Source: Oncotarget. 2017 Feb 18;8(13):21115–27. doi: 10.18632/oncotarget.15501 (PMC5400570; doi:10.18632/oncotarget.15501)
Supplement: Supplementary file 1 [file oncotarget-08-21115-s001.pdf]

Acute lymphoblastic leukemia cells are sensitive to disturbances in protein homeostasis induced by proteasome deubiquitinase inhibition

Supplementary Materials

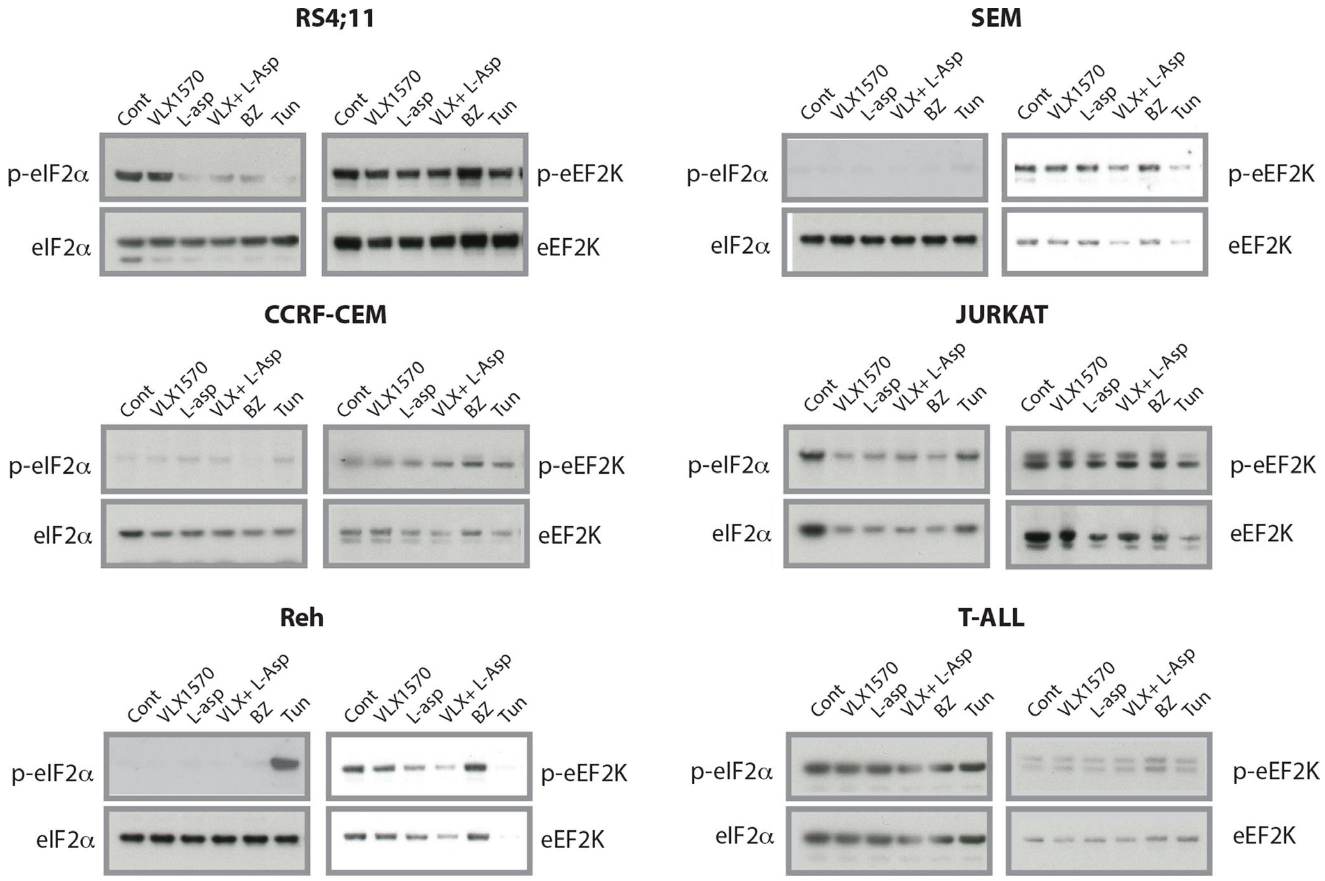

Supplementary Figure 1: ALL cells were exposed to VLX1570 and/or L-Asp or bortezomib (BZ) for 6 hours. Protein lysates were subjected to immunoblotting with antibodies to p-eIF2α, eIF2α, p-eEF2K or eEF2K. Tunicamycin was used as a refence drug to induce ER stress.
